# Supplementary material for: Protein Structure Validation and Refinement Using Amide Proton Chemical Shifts Derived from Quantum Mechanics
Source: PLoS One. 2013 Dec 31;8(12):e84123. doi: 10.1371/journal.pone.0084123 (PMC3877219; doi:10.1371/journal.pone.0084123)
Supplement: Supporting Information S1 — Section S1: Time evolution of energies and chemical shift RMSDs during MCMC simulation. Figures S1–S3: Details of Monte Carlo energies and chemical shift RMSDs over time for the presented simulations. Section S2: Parametrization of chemical shift contributions due to hydrogen bonding interactions to carboxylic acids and alcohols. Figure S4: Sketches showing the geometric parameters and the systems used in the modeling of chemical shift contributions due to hydrogen bonding. Section S3: Model for solvent exposed amide protons. Table S1: Chemical shift contributions due to hydrogen bonding to water molecules. Figure S5: Local minima of NMA-water dimer. (PDF) [file pone.0084123.s001.pdf]

S1 Time evolution of energies and chemical shift RMSDs

A: SMN Tudor Domain

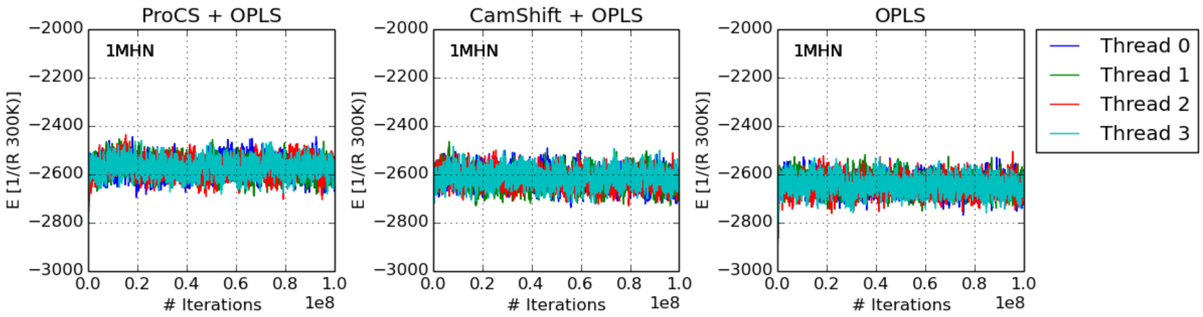

B: Protein G

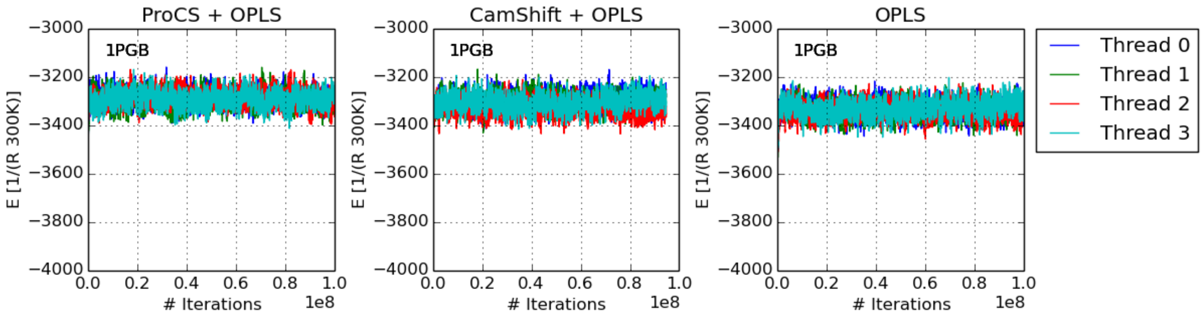

C: Ubiquitin

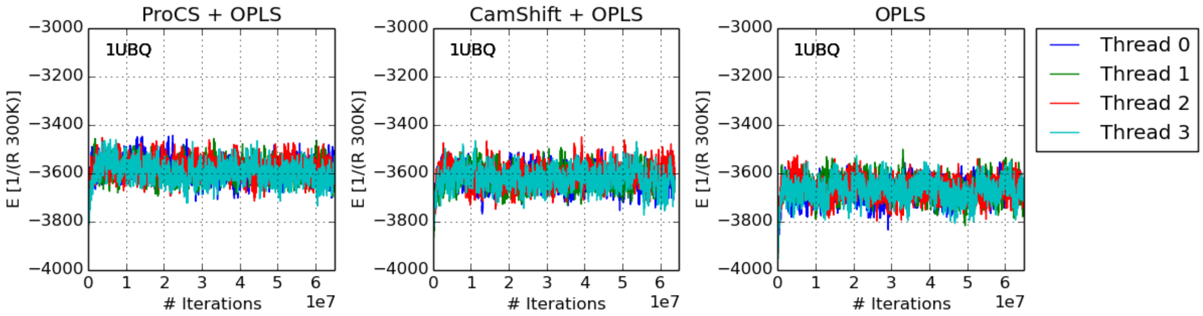

Figure S1: Monte Carlo energy over time.

A: SMN Tudor Domain

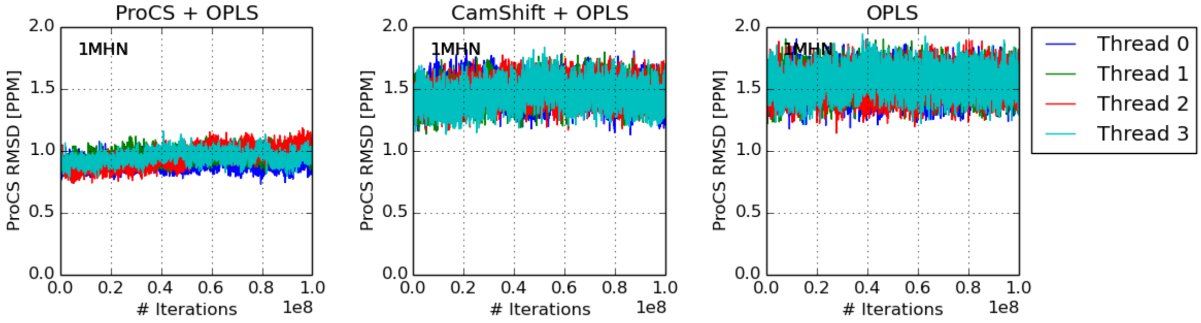

B: Protein G

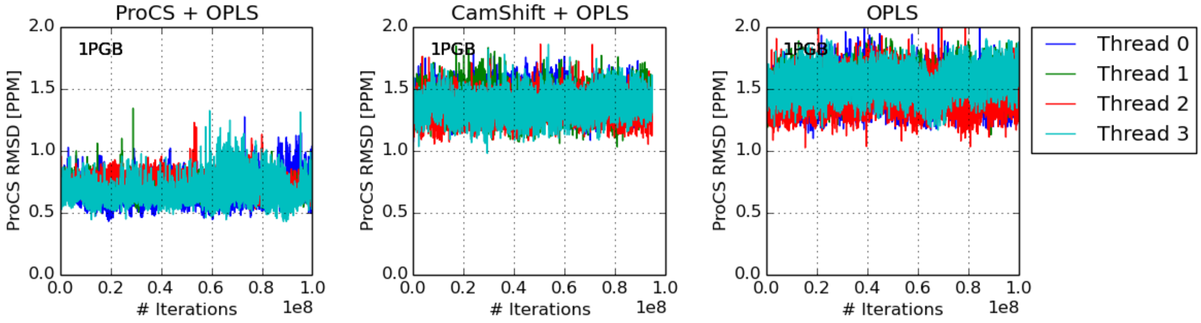

C: Ubiquitin

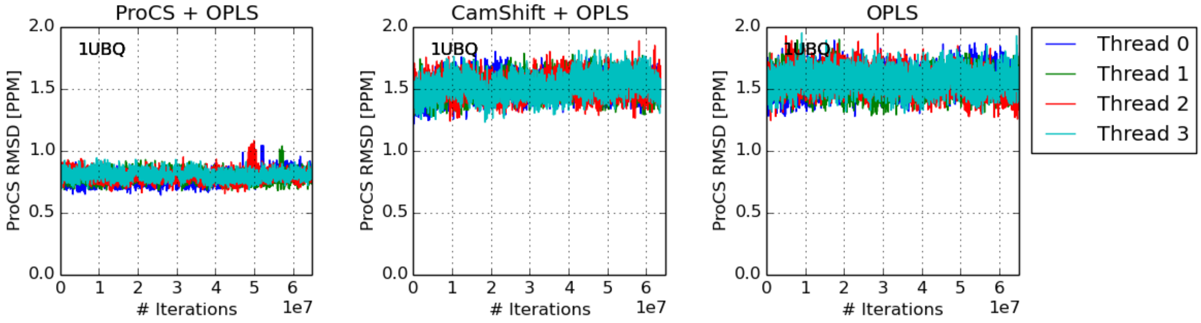

Figure S2: ProCS calculated chemical shifts RMSD to experimental chemical shifts over time

A: SMN Tudor Domain

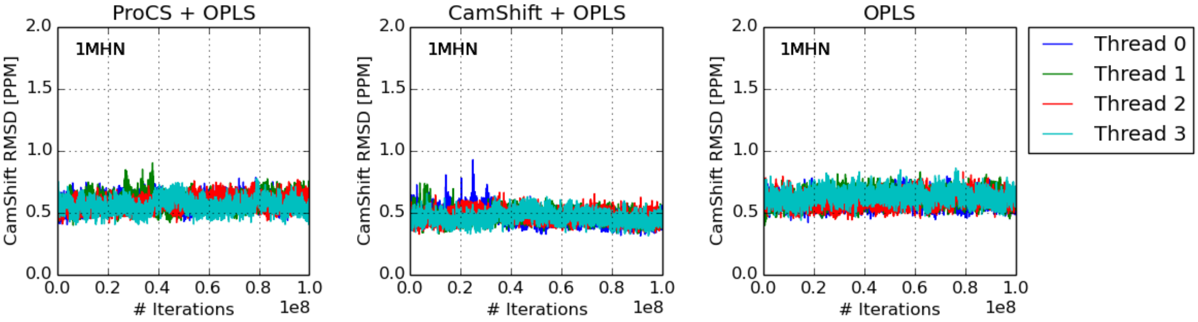

B: Protein G

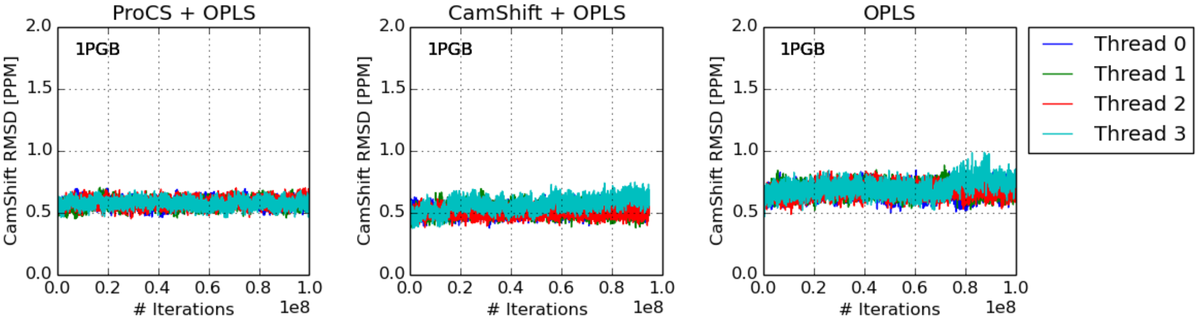

C: Ubiquitin

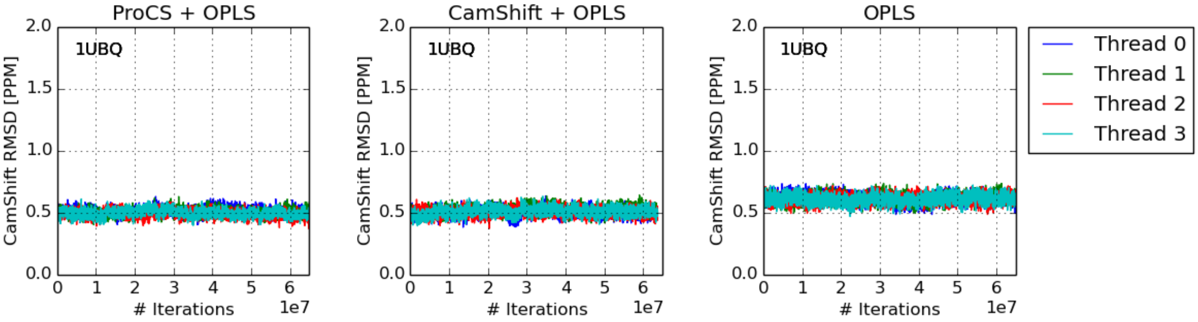

Figure S3: CamShift calculated chemical shifts RMSD to experimental chemical shifts over time

S2 Bonds to carboxylic acids and alcohols

In this section, a model of the chemical shift contributions due to hydrogen bonding to carboxylic acids and alcohol side chains are described. These are common hydrogen bonding acceptors in proteins. Since the C-terminus and the aspartic acid and glutamic acid side chains are, in the current `Phaistos` framework, always given in the deprotonated state, only this state is considered in this section.

An approach similar to the amide-amide model due to Barfield[1] is used. The model system consists of two molecules, modeling the amide hydrogen bonding donor and acceptor complex, and the geometric dependence is modeled by scanning conformations over relevant angles and distances.

As an approximation to the carboxylic acid functional groups found in the protein (aspartate, glutamate and the C-terminus), an acetate anion is used. The alcohol functional groups, threonine, serine and tyrosine are approximated by a methanol. As a backbone amide model, an *N*-methylacetamide (NMA) molecule is used.

Using minimal amide, carboxylic acid and alcohol models, a scan over a range of bond angles and distances is carried out. The hydrogen bonding distance is modeled in a range from 1.5 Å to 2.5 Å in 0.125 Å steps. Shorter hydrogen bonds are unlikely and the cutoff used in determining the presence of a hydrogen bond in this work is 2.5 Å. The dihedral angles H..O=C-C and H..O-C(..)H^{O} are scanned over the full 360° range in 15° intervals. Finally, the H..O-C angle was scanned from 180° to 90° in 10° intervals. To avoid steric clashes between the methyl groups of the NMA molecule and the methanol/acetate, the N-H..O bond angle was fixed at 180°. See Fig. S4 for an overview of the models. The size of both grids were 1944 model systems, for which the NMR shieldings was calculated.

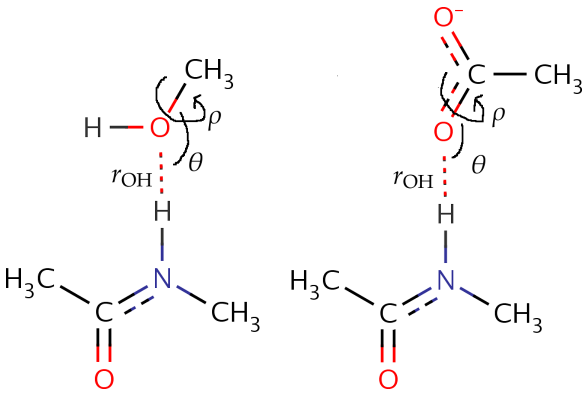

(A) **(B)**

Figure S4: Sketches showing the geometric parameters and the systems used in the modeling of chemical shift contributions due to hydrogen bonding. In A), the θ angle is defined as the H..O-C angle, while ρ is defined as the H..O-C(..)H^{O} dihedral angle. In B), θ is the H..O-C angle, and ρ is the H..O=C-C dihedral.

To allow for the prediction of any geometry between the grid points, a linear interpolation algorithm is used.

S3 Solvent exposed amide protons

Accurate modelling of solvent around the protein is an extremely difficult problem in modern computational chemistry due to the complexity involved. Since `Phaistos` generate structures, that do not contain

explicit solvents (e.g. crystallographic water molecules), the simplistic model used in this work implicitly assumes the presence of water molecules, when the amide proton is considered to be solvent exposed.

The chemical shift contribution from solvent exposure of the amide proton is here assumed to be equivalent to the contribution from a hydrogen bond to a water molecule. A typical contribution from a water molecule in a energy minimized hydrogen bonding conformation is found in section S3.1 to be +2.07 ppm. As a crude approximation solvent exposed amide protons are assigned with a fixed +2.07 ppm primary bond contribution.

S3.1 Hydrogen bonding to a water molecule

A water molecule is placed near the amide hydrogen atom of a probe NMA molecule and a B3LYP/6-311++G(d,p) minimization is carried out. The resulting structure is a local minimum of an amide hydrogen bonded to a water molecule. From this geometry the chemical shift of the entire dimer is then calculated.

Using four different starting geometries, the water molecule was minimized into two different conformation (see Fig. S5A and S5B). The hydrogen bonding geometry of these two conformations had a few similarities. The water oxygen was in both cases aligned into the N-H bond axis. The hydrogen bonding distance was also similar at 2.04 Å and 2.07 Å, respectively. To separate the change in chemical shift due to change of the internal geometry of the NMA molecule, another NMR calculation was carried out using the optimized systems, but with the water molecule removed. One minimization done without the geometry restriction led to a 60° rotation of a methyl group. The subsequent analysis of the systems with the water molecule removed revealed that the rotation caused an extra shielding of about 0.2 ppm. However, by using the NMA geometries from the optimized dimer as a reference, this artifact was removed. The resulting chemical shift due to the water molecule turned out to be very similar, at +2.04 ppm and +2.09 ppm respectively. The chemical shift in these minima serve as rough figures for the chemical shift due to solvent exposure. We thus assign a contribution of +2.07 ppm to the total chemical shift of solvent exposed amide protons.

Chemical Shift	Minimum A	Minimum B
Optimized NMA-Water Dimer	6.23 ppm	6.45 ppm
Optimized NMA alone	4.19 ppm	4.37 ppm
Difference	+2.04 ppm	+2.09 ppm

Table 1: The chemical shift of the amide proton in two local energy minima. "NMA-Water Dimer" is the chemical shift of the NMA amide proton in the dimer. "NMA alone" is the amide proton chemical shift is the resulting chemical shift of the NMA amide proton in the optimized configuration when the water molecule is removed and no furter optimization is carried out. The resulting difference corresponds to the change in chemical shift due a hydrogen bond to a water molecule.

References

[1] Barfield M (2002) Structural Dependencies of Interresidue Scalar Coupling ${}^{h3}J_{NC'}$ and Donor ^{1}H Chemical Shifts in the Hydrogen Bonding Regions of Proteins. J Am Chem Soc 124:4158-4168.

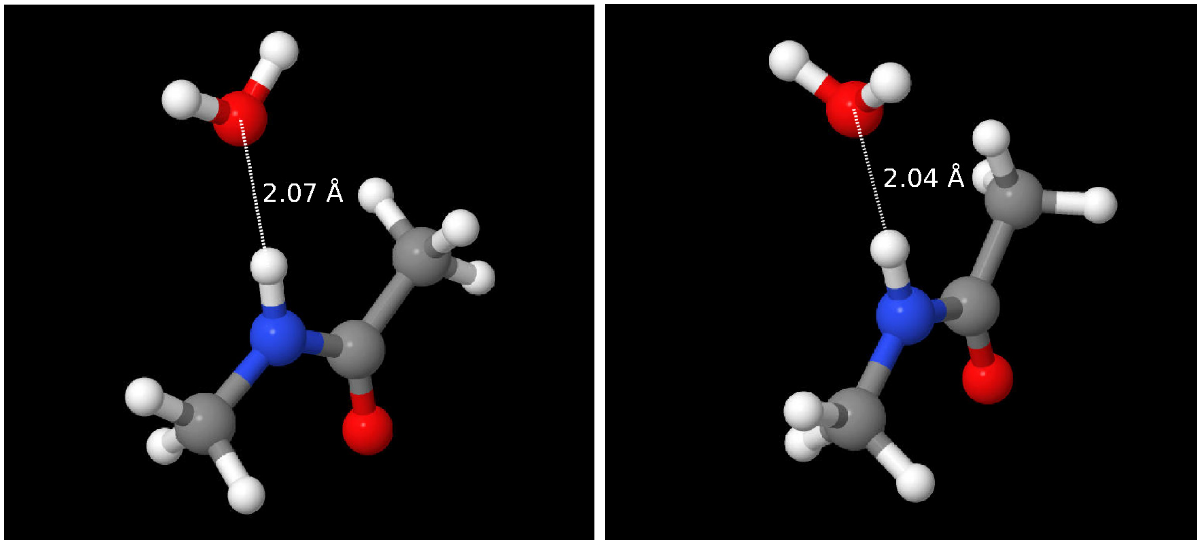

(A) **(B)**

Figure S5: Two different local energy minima of the NMA-water dimer. The hydrogen bonding distances are almost identical. The water molecule is rotated 90° between A and B, and one methyl group has a 60° difference in rotation between A and B.
